# Health Workers’ Knowledge and Attitude towards Monkeypox in Southwestern Saudi Arabia: A Cross-Sectional Study

**DOI:** 10.3390/diseases11020081

**Published:** 2023-06-02

**Authors:** Nasser H. Sobaikhi, Najim Z. Alshahrani, Rakan S. Hazazi, Hafiz I. Al-Musawa, Raed E. Jarram, Amjad E. Alabah, Nawaf F. Haqawi, Fadi A. Munhish, Mohammed A. Shajeri, Mohammed H. Matari, Riyadh M. Salami, Alhassan H. Hobani, Najla A. Yahya, Abdulaziz H. Alhazmi

**Affiliations:** 1 Faculty of Medicine, Jazan University, Jazan 45142, Saudi Arabia; 2 Faculty of Medicine, University of Jeddah, Jeddah 21589, Saudi Arabia; 3 Faculty of Pharmacy, Jazan University, Jazan 45142, Saudi Arabia

**Keywords:** monkeypox, mpox, knowledge, attitude, health workers, Saudi Arabia, Jazan

## Abstract

Background: Monkeypox outbreaks in non-endemic countries emphasize the importance of being prepared to prevent its progression to a pandemic. To effectively control monkeypox, healthcare providers must have sufficient knowledge and good attitudes and practices to limit its spread. We initiated this project to assess the factors associated with health workers’ knowledge and attitude toward monkeypox in southwestern Saudi Arabia. Methods: We included 398 eligible health workers working at various health facilities. Data was collected using an online survey, and participants had an opportunity to consent. We conducted descriptive statistics for all variables and used chi-square statistics, *t*-test, and multivariate analysis to establish the association between health workers’ demographic characteristics and knowledge of monkeypox disease. Results: The mean age was 30.93 ± 8.25 years for the included participants, and most of them were between 22 and 29 years, male, single, nurses, working in government hospitals, and had worked for at least five years. The chi-square and *t*-test showed that the participants’ knowledge level was significantly related to age, marital status, job title, and medical practice. Most of the participants had low knowledge and good attitudes toward monkeypox prevention measures. Multivariate analysis showed that higher knowledge was associated with younger age after controlling all other significant bivariate relationships between knowledge and demographics. Conclusions: This study found low knowledge levels and high good attitude levels of monkeypox among the participants. As such, there is a need to support health workers in understanding monkeypox epidemiology, prevention, and treatment. Therefore, Saudi Arabia will be making significant strides to being well prepared and ready to handle future monkeypox outbreaks.

## 1. Introduction

The European Union, the United Kingdom (UK), North America, and Australia experienced a monkeypox outbreak in 2022 [1]. Given the global burden of this outbreak, the World Health Organization (WHO) declared monkeypox an international public health emergency in 2022, calling all countries to take measures to curb the spread of the disease. Several countries adopted various infection prevention measures to control the disease. Some infection control measures included screening and isolation of people, hand hygiene, use of personal protective equipment, cleaning and disinfecting surfaces, and safe management of hospital wastes [2].

Monkeypox is a zoonotic disease caused by the monkeypox virus that belongs to the Orthopoxvirus genus. This virus spreads mainly from infected animals to humans [3,4,5,6]. Infected humans can also spread the disease to an uninfected human through respiratory secretions, infected skin lesions, and any other contaminated materials [2,4,5,6,7]. Monkeypox disease has a case fatality rate of between 1% and 10% and is commonly characterized by skin rashes on the face and arms, fever, inguinal lymphadenopathy, weakness, fatigue, malaise, and headache [1,3,4,5,6].

The distinguishing feature of monkeypox from smallpox is lymphadenopathy. Monkeypox manifests similarly to smallpox and the smallpox vaccine can be protective by up to about 85% [3,4,8]. Monkeypox also similarly manifests as chickenpox, and the prominent distinctive feature is the superficial and clustered lesions in the palms and soles in severe chickenpox [8]. When monkeypox is not identified early and properly managed, it can result in harmful effects such as secondary bacterial infections, respiratory distress, bronchopneumonia, encephalitis, and ocular diseases [8].

While monkeypox might not be of high concern now, recent trends showed that there is a need for Middle Eastern countries’ health professionals to be well prepared to prevent the disease [1,7]. Evidence shows that monkeypox has increased over tenfold in Africa between 1970 and 2022, and there have been erratic outbreaks outside Africa in the last eight years [9,10]. Although Middle Eastern countries have not declared outbreaks, some countries reported monkeypox cases. However, following the 2022 global public health emergency, Middle Eastern countries reported 249 monkeypox cases, and Saudi Arabia had already registered 5 confirmed cases by August 2022 [1,9].

For any international public health emergency, such as the monkeypox outbreak in 2022, all nations must have immediate outbreak response plans for timely disease prevention. Alongside other emergency response materials and equipment, local governments need to inform all people of the signs and symptoms of the disease [2,10]. All health workers should be educated about monkeypox through continuous health education or short-term emergency training courses [1,2]. Health officials should strengthen public health and disease surveillance capacities in Middle Eastern countries to effectively mitigate monkeypox. This is particularly important given that Saudi Arabia experienced a Rift Valley fever outbreak in 2000 in the Jazan Province, resulting in 500 cases and many deaths [11].

Unfortunately, no evaluative studies have been conducted to understand the extent to which Saudi Arabia is prepared to respond to any monkeypox disease outbreak. Alsharani et al. have explored the knowledge among the general population, including general practitioners, about the disease [7]. The study surveyed 1212 participants from various Saudi Arabian regions using a self-administered questionnaire. The participants’ overall knowledge about monkeypox viral infection was low, with only 20.4% of respondents having heard of the disease. The study also compared the general population’s knowledge to that of general practitioners in Saudi Arabia. It showed that the general practitioners had a significantly higher level of knowledge about monkeypox viral infection than the general population. As such, there was a need to understand the potential factors that may affect such knowledge and attitude from the perspective of all the health worker professions. Therefore, this study aimed to assess the factors associated with health workers’ knowledge and attitude toward monkeypox in southwestern Saudi Arabia.

## 2. Material and Methods

### 2.1. Study Design and Setting

We conducted a cross-sectional descriptive study among health workers in the southwestern region of Saudi Arabia from 4 November 2022 to 8 December 2022. The study participants were health practitioners practicing in various healthcare facilities in the southwestern region of Saudi Arabia at the time of the study. These participants were recruited through a convenience sampling technique. Health practitioners that consented to participate were included in the research, and only completed questionnaires from health workers were considered in this study. We calculated the sample size for this study to be 384 participants using http://www.raosoft.com/samplesize.html (Raosoft Inc., Seattle, WA, USA, accessed on 1 February 2023) with a 95% confidence interval, an error rate of less than 5% with a prevalence of 50%.

### 2.2. Data Tool and Collection

An online survey in Arabic was sent to health practitioners who practice in healthcare facilities in the southwestern region of Saudi Arabia. A pretested survey was uploaded on Google Form templates and sent to participants by email and social media platforms (WhatsApp and Twitter) [7]. The knowledge part of the survey was partly modified to meet study objectives. To address the validity of responses, we conducted a pilot study involving twenty physicians to pretest our survey instrument. Internal consistency was evaluated using Cronbach’s alpha, which yielded a value of 0.86. The survey was divided into four sections: sociodemographic, medical practice experience, monkeypox knowledge, and attitudes toward monkeypox sections (Table 1). Sociodemographic characteristics, comprising age, marital status, job type, work sector, and experience were studied. The examination of monkeypox knowledge was limited to individuals who had prior awareness of the disease. Before completing the responses, each respondent was asked for their informed consent by clicking the consent statement. The informed consent statement described the study’s purpose and objectives in detail. As previously described [7], knowledge scores were categorized into low and high for the scoring system. We used the mean score of 14 ± 5 as a cut-off point. A mean score > 14 was denoted as high knowledge, and 14 and less was considered low knowledge. To investigate the predictors of monkeypox knowledge, we categorized participants into two groups based on their knowledge scores: high and low. Sociodemographic characteristics, including age, marital status, job type, work sector, and experience, were then used as independent variables to examine their associations with higher levels of monkeypox knowledge.

### 2.3. Data Analysis

Statistical Package for Social Science (SPSS) version 26 (IBM Corp., Armonk, NY, USA) was used for data analysis. Frequencies were obtained for all sociodemographic variables. Chi-square and *t*-test statistics were also conducted to understand the relationship between sociodemographic variables and the participants’ knowledge scores. For the alpha criterion for the *p*-value, it was set to 0.05. Multivariate analysis was performed using multiple logistic regression for variables significantly associated with knowledge in univariate analysis.

### 2.4. Ethical Considerations

Our study was conducted according to the Declaration of Helsinki. Ethical approval was sought from the Research Ethics Committee (REC) at Jazan University, and the approval number is REC-44/03/321. Participants were assured of their information confidentiality if they chose to respond to the survey.

## 3. Results

As indicated in Table 1, 398 health workers with an average age of 30.93 ± 8.25 years participated in this study. The majority of these participants were males (61.8%), single (51%), nurses (32.4%), government workers (85.9%), and with more than five years of work experience (45.7%). Most participants (86.7%) indicated having heard about monkeypox before the study, and only 13.3% had not heard about it. The major source of information about monkeypox for these participants was social media (68.8%).

Table 2 shows the participants’ knowledge regarding monkeypox. The majority of the participants knew monkeypox was prevalent in Western and Central Africa (55%) but not in the Middle Eastern countries (53.3%) and were not even aware of any human monkeypox cases in Saudi Arabia (86.9%). Regarding the type of disease, most participants correctly identified monkeypox as a viral disease (78.9%) and not bacterial (65.6%). Although most participants (58.8%) knew that monkeypox could be transmitted from human to human, many (77.9%) did not understand that a bite from an infected monkey could also spread it. Most participants did not know monkeypox had similar signs and symptoms as chicken pox (85.2%) and smallpox (91.2%). While the majority of the participants knew skin rashes (72.9%), papules (52.5%), vesicles (54.8%), and pustules (57.5%) as signs and symptoms of monkeypox, an equally considerable number did not know that flu-like syndrome (52.3%), lymphadenopathy (58%), and diarrhea (77.1%) were also signs and symptoms of monkeypox. Regarding the management of monkeypox, almost the same proportion of participants reported using viral drugs (52.3%) and antibiotics (64.8%) to fight against monkeypox. Most participants also incorrectly reported paracetamol as a management option for monkeypox (54.3%) and stated that infected people could get vaccinated (65.3%). Most participants incorrectly reported monkeypox to have a specific vaccine (82.2%) and treatment (53.8%).

Participants that correctly responded to the questions were with good knowledge and those who incorrectly responded were with low knowledge regarding monkeypox infection according to the previously described scoring system (Table 2). As such, Table 3 below shows the chi-square and *t*-test statistics for the association between participants’ sociodemographic characteristics and knowledge categorization (high versus low knowledge) among participants who had heard about monkeypox (n = 345). The *t*-test and chi-square statistics show that age, marital status, job title, and medical practice experience were statistically significant.

The younger participants were more likely to have high knowledge of the monkeypox infection. Further, unmarried participants were more likely than married participants to have high knowledge of monkeypox. The nurses were more likely to have high knowledge of the monkeypox disease than physicians, while the health workers who had worked for less than one year were more likely than those who had stayed longer to have high knowledge of monkeypox. Multivariate analysis showed that only younger age was significantly associated with high knowledge of monkeypox (*p*-value: 0.046, aOR = 1.052, 95% C.I. = 1.001–1.107) when all other significant bivariate relations between the demographics variables and knowledge were controlled.

Table 4 shows the participants’ responses to attitudes questions about the monkeypox virus, emerging diseases, and travel epidemiology. As indicated in this table, the attitudes of the majority of the participants toward the different aspects of the monkeypox virus were good. The majority believed that the virus could be controlled worldwide (63.3%), that the Saudi Ministry of Health could control the virus (76.9%), and that mass media could influence the worldwide prevention of the disease (73.1%). Regarding containing the disease, most participants disagreed that monkeypox disease has the potential to be pandemic (37.9%). However, they agreed that the disease could be a new burden to the healthcare systems of the affected country (55.3%). Most importantly, the majority of the participants wanted to learn about the monkeypox infection (74.6%), the epidemiology of new emerging diseases (75.4%), and travel medicine (71.1%).

## 4. Discussion

The monkeypox outbreak burden has been reportedly high in Europe, the UK, North America, and Australia. However, with the evidence that the frequency and severity of such outbreaks has dramatically increased, all countries must be prepared to respond to any future occurrences effectively and adequately [10]. The WHO has subsequently guided preparedness, readiness, and response in this regard through five cores of emergency coordination, collaborative surveillance, community protection, safe and scalable care, and countermeasure and research [2]. At the center of these five core components is the health workers’ knowledge of monkeypox and their attitude toward the disease and its related response measures. Sirwan et al. assert that all health employees should be educated on monkeypox, increasing their knowledge and attitude toward monkeypox outbreak response, prevention, and readiness [1].

Our study shows that most participants knew monkeypox as a viral disease, but could not succinctly articulate all its transmission modes, signs and symptoms, treatment, and vaccination appropriateness. While one might be moved to believe that health workers know these aspects of monkeypox disease, our study indicated otherwise. There is a need to adequately support health workers to be knowledgeable regarding monkeypox transmission and prevention. Our findings agreed with other studies from Saudi Arabia and Bangladesh among medical doctors [7,12]. In Saudi Arabia, physicians had low knowledge of the prevalence of monkeypox, its transmission, and clinical differences with other similar diseases, such as smallpox, chickenpox, and influenza. In Bangladesh, only 31% of the medical doctors correctly answered at least 70% of the questions [13]. The observation of poor monkeypox knowledge among health workers might suggest that local health officials in Saudi Arabia have done less to equip health workers with appropriate formal information related to monkeypox. This assertion is evidenced by the fact that additional results in the current study showed that although most health workers had heard about monkeypox, their primary source of information was social media. Low knowledge levels of monkeypox disease among health workers elsewhere have mainly been associated with the lack of information documentation and not teaching the disease in medical and nursing schools, which also apply to Saudi Arabia [12]. As far as monkeypox preparedness, readiness, and prevention are concerned, there is a need for intentional efforts by the Saudi Ministry of Health to educate its health workers on all emergency-related diseases. The Ministry of Education might also be needed to equip medical and nursing students with monkeypox disease information as part of the essential endemic diseases.

Additionally, among other factors, higher knowledge of monkeypox was significantly associated with the participants’ age, marital status, job title, and practice experience in univariate analysis. A study by Alshahrani et al. agrees with our study on age findings and indicates no significant results on the factor of practice experience [7]. While there are no existing studies to explain the interaction of these factors with monkeypox knowledge, it can be assumed that younger health workers are more interested in updating themselves on several aspects of health matters, especially where there is no official effort to extend appropriate information to the health workers compared to their older counterparts. Consequently, it is essential to note that young participants aged between 22 and 29 made up more than half of the total participants. As such, they could have confounded this significant observation. However, multivariate analysis showed that only younger age is significantly associated with higher knowledge of monkeypox when all other significant bivariate relations between the demographics variables and knowledge are controlled.

Given that no specific training has been conducted for any health profession, nurses have been observed to possess a better knowledge of monkeypox than physicians in univariate analysis. This finding may deserve further investigation. Despite it is expected that the more years in practice, the more one is exposed to most diseases, including monkeypox. However, health workers in the current study who had stayed for more than five years were found to have lower knowledge of monkeypox than those who had stayed for fewer years in univariate analysis by the time of the study. This finding could be explained by the motivation of new employees in medical fields to be updated as the disease has become more well-known and studied in recent years. Moreover, newer workers may have had more exposure to information about monkeypox during their medical training, as medical schools and residency programs have likely updated their curricula to include more information about emerging infectious diseases.

While knowledge level affects attitude level, it was not part of the scope of this study to test this association. However, our results showed that participants had low monkeypox knowledge yet good attitudes towards monkeypox aspects. Hasan et al. tested the association between participants’ knowledge and attitude toward monkeypox and its preventive measures and showed that 85% of doctors had positive attitudes and only 31% of participants possessed good knowledge regarding monkeypox [12]. Participants in our study believed the disease could be controlled worldwide and by health officials. They also recognized the role mass media play in influencing disease prevention. Most participants wanted to learn about infection, epidemiology, and travel medicine related to monkeypox. The health belief model asserts that people’s beliefs influence their health-related actions [13]. As such, health workers believe that learning about monkeypox is important, and their interest in learning is critical to changing their behaviors toward and understanding of monkeypox disease and its response dimensions. Therefore, health officials should be able to ride on smooth grounds to prepare the health workers for monkeypox disease prevention.

Further, this is particularly important given the history of outbreaks in the Jazan province of Saudi Arabia that were imported from Africa, as happened with the Rift Valley fever outbreak [11,14,15,16]. While no outbreaks of monkeypox have been reported in Saudi Arabia to date, the risk of importation cannot be ignored, given the high volume of international travel in the region, especially during religious seasons [17,18,19,20]. There have also been outbreaks of other infectious diseases of zoonotic origin in Saudi Arabia and Jazan, including MERS-CoV, SARS-CoV-2, dengue fever, and others, highlighting the need for effective disease surveillance and response capacity [21,22,23,24,25,26,27]. By providing targeted training and educational programs, health officials can ensure that health workers have the knowledge and skills to prevent and control outbreaks effectively. This will protect the health and safety of the community and help prevent the spread of disease to other regions and countries [28,29,30].

This study is crucial in supporting the country in understanding public health emergency areas that need strengthening. While in this study, we focused on all health worker professions, which was not the case in the previous studies. However, this study has been limited by the non-randomness of the study design, and our survey was self-reported; thus, it is liable to information bias and may bear variables that confounded with each other despite the efforts to limit this confounding effect. Thus, despite being significant in univariate analysis, marital status, job title, and medical practice seem to be cofounders, and only age is considered a significant predictor variable of knowledge of monkeypox. Additionally, it was performed via specific online platforms, which denotes that we may not have contacted subgroups of this population, such as other healthcare workers who do not utilize these platforms. Although our study provides useful insights into the knowledge of monkeypox among healthcare workers, it is important to note that a formal validation study was not conducted for the modified tool used to test knowledge of monkeypox, which may limit the generalizability of our findings and affect the reliability of the tool. Further, the potential influence of demand characteristics cannot be fully ruled out, as the wording and framing of the introductory statement could have potentially influenced how participants responded to the survey items. Another point that should be considered in the questionnaire is that the answers to some questions may not be evident, such as questions 16 and 22. However, we believe this will not affect the knowledge score of the participants. Therefore, the findings cannot be generalized, and nationwide studies may be appropriate for searching other aspects related to this topic. Moreover, qualitative research should be administered to obtain a reasonable, broad, and more thorough understanding of the knowledge and attitude of human monkeypox viral infection among healthcare workers in Saudi Arabia.

## 5. Conclusions

In this study, we investigated the attitudes and knowledge of health workers in the Jazan province, Saudi Arabia, towards monkeypox disease and its prevention. Our findings indicate that while the overall attitude toward the disease and its prevention is good, the knowledge level among health workers is low, which is a concerning issue, as accurate knowledge about the disease is crucial for effectively preventing and controlling outbreaks. Our univariate analysis also revealed that age, marital status, job title, and medical practice experience are significant factors associated with monkeypox knowledge among health workers. Notably, younger age was significantly associated with higher knowledge in multivariate analysis when all other significant bivariate relations between the demographics variables and knowledge were controlled. These findings suggest that targeted training and educational programs should be developed and implemented, particularly for older health workers who may have received less formal education about the disease. Importantly, our study also highlights the need for increased education and awareness about monkeypox disease among health workers in the Jazan province. While our participants indicated a strong desire to learn about the disease, its epidemiology, and related travel medicine, we found that local officials have not done enough to educate health workers about it. This lack of education could spread falsified information about the disease, which could have severe consequences in the event of an outbreak. By taking these steps, we can better prepare for future outbreaks and ensure the health and safety of the community.

## Figures and Tables

**Table 1:** Sociodemographic characteristics of participants (n = 398).

Variables	Frequency	Percent (%)
Age (years) Mean|SD	30.93	8.25
Gender		
Male	246	61.8
Female	152	38.2
Marital status		
Unmarried	212	53.3
Married	186	46.7
Job title		
Physician	58	14.6
Nurse	129	32.4
Laboratory	51	12.8
Other health specialties	160	40.2
Work sector		
Governmental	342	85.9
Private	56	14.1
Medical practice experience		
Less than one year	121	30.4
1–5 years	95	23.9
More than five years	182	45.7
I have heard about monkeypox before		
Yes	345	86.7
No	53	13.3
Source of information about monkeypox		
Medical books or during my studies	33	8.3
Social media	274	68.8
Healthcare workers	38	9.5

**Table 2:** Participants’ responses to knowledge questions about the monkeypox virus.

Knowledge Questions	Correct- N (%)	Incorrect- N (%)
Q1. Is monkeypox prevalent in Middle Eastern countries? (Answer: No)	186 (46.7)	212 (53.3)
Q2. Is monkeypox prevalent in Western and Central Africa? (Answer: Yes)	219 (55.0)	179 (45.0)
Q3. Are there any human monkeypox cases in Saudi Arabia? (Answer: Yes)	52 (13.1)	346 (86.9)
Q4. Is monkeypox a viral disease infection? (Answer: Yes)	314 (78.9)	84 (21.1)
Q5. Is monkeypox a bacterial disease infection? (Answer: No)	261 (65.6)	137 (34.3)
Q6. Is monkeypox easily transmitted human-to-human? (Answer: Yes)	234 (58.8)	164 (41.2)
Q7. Could monkeypox be transmitted through a bite of an infected monkey? (Answer: Yes)	88 (22.1)	310 (77.9)
Q8. Are travelers from America and Europe the primary source of imported cases of monkeypox? (Answer: No)	198 (49.7)	200 (50.3)
Q9. Do monkeypox and smallpox have similar signs and symptoms? (Answer: Yes)	36 (9.0)	362 (91.0)
Q10. Do monkeypox and chickenpox have similar signs and symptoms? (Answer: Yes)	59 (14.8)	339 (85.2)
Q11. Is a flu-like syndrome one of the early signs or symptoms of human monkeypox? (Answer: Yes)	190 (47.7)	208 (52.3)
Q12. Are rashes on the skin one of the signs or symptoms of human monkeypox? (Answer: Yes)	290 (72.9)	108 (27.1)
Q13. Are papules on the skin one of the signs or symptoms of human monkeypox? (Answer: Yes)	209 (52.5)	189 (47.5)
Q14. Are vesicles on the skin one of the signs or symptoms of human monkeypox? (Answer: Yes)	218 (54.8)	180 (45.2)
Q15. Are pustules on the skin one of the signs or symptoms of human monkeypox? (Answer: Yes)	229 (57.5)	169 (42.5)
Q16. Is diarrhea one of the signs or symptoms of human monkeypox? (Answer: Yes)	91 (22.9)	307 (77.1)
Q17. Is lymphadenopathy (swollen lymph nodes) one clinical sign or symptom that could be used to differentiate between monkeypox and smallpox cases? (Answer: Yes)	167 (42.0)	231 (58.0)
Q18. One management option for symptomatic monkeypox patients is to use paracetamol. (Answer: No)	182 (45.7)	216 (54.3)
Q19. Are antivirals required in the management of human monkeypox patients? (Answer: No)	208 (52.3)	190 (47.7)
Q20. Are antibiotics required in the management of human monkeypox patients? (Answer: No)	140 (35.2)	258 (64.8)
Q21. Are people who got the chickenpox vaccine immunized against monkeypox? (Answer: Yes)	138 (34.7)	260 (65.3)
Q22. Is there a specific vaccine for monkeypox? (Answer: No)	71 (17.8)	327 (82.2)
Q23. Is there a specific treatment for monkeypox? (Answer: No)	184 (46.2)	214 (53.8)

**Table 3:** Association between participants’ sociodemographic variables and knowledge scores (n = 345).

Variables	High Knowledge (n = 152, 44.1%)	Low Knowledge (n = 193, 55.9%)	*p*-Value
Age in years (Mean|SD)	28.97|6.46	32.71|8.38	0.0001 *
Sex			
Male	95 (62.5)	121 (62.7)	1.000
Female	57 (37.5)	72 (37.3)	
Marital status			
Unmarried	90 (59.2)	79 (40.9)	0.0001 *
Married	63 (41.1)	119 (61.6)	
Job title			
Physician	14 (9.2)	39 (20.2)	0.044 *
Nurse	54 (35.5)	60 (31.1)	
Laboratory	22 (14.5)	22 (11.4)	
Other health specialties	62 (40.8)	72 (37.3)	
Sector			
Governmental	129 (84.9)	167 (86.5)	0.756
Private	23 (15.1)	26 (13.5)	
Experience			
Less than 1 year	59 (38.8)	48 (24.9)	0.002 *
1–5 years	39 (25.7)	41 (21.2)	
More than 5 years	54 (35.5)	104 (53.9)	
When did you hear about monkeypox?			
Within several days or weeks ago	16 (10.5)	16 (8.3)	0.576
Within the last month or later	136 (89.5)	177 (91.7)	
Source of information about monkeypox			
Medical books or during my studies	12 (7.9)	21 (10.9)	0.512
Social media	121 (79.6)	153 (79.3)	
Healthcare workers	19 (12.5)	19 (9.8)	

* Significant when the alpha criterion for *p*-value was set to 0.05.

**Table 4:** Participants’ responses to attitudes questions about the monkeypox virus, emerging diseases, and travel epidemiology (n = 398).

Sentence	Agree n (%)	No Opinion n (%)	Disagree n (%)
I am confident that the world’s population can control monkeypox worldwide.	252 (63.3)	95 (23.9)	51 (12.8)
I am confident that the Saudi MOH and local population can control monkeypox locally.	306 (76.9)	60 (15.1)	32 (8)
I have bad feelings toward the monkeypox virus that it might become a worldwide pandemic.	117 (29.4)	130 (32.7)	151 (37.9)
I think that monkeypox can add a new burden on the healthcare system of the affected countries.	220 (55.3)	114 (28.6)	64 (16.1)
I think monkeypox can be transmitted to Saudi Arabia	227 (57)	128 (32.2)	43 (10.8)
I think that mass media coverage of monkeypox may influence its worldwide prevention.	291 (73.1)	71 (17.8)	36 (9)
I want to learn more about monkeypox.	297 (74.6)	68 (17.1)	33 (8.3)
I want to learn more about the epidemiology of the new emerging diseases.	300 (75.4)	64 (16.1)	34 (8.5)
I want to learn more about travel medicine.	283 (71.1)	73 (18.3)	42 (10.6)
I think that it is dangerous to travel to the countries epidemic with monkeypox.	285 (71.6)	76 (19.1)	37 (9.3)

## Data Availability

Data are available upon a reasonable request from the corresponding author.
